# Level of insulin adherence among diabetes mellitus patients in Felege Hiwot Referral Hospital, Bahir Dar, Northwest Ethiopia, 2017: a cross-sectional study

**DOI:** 10.1186/s13104-018-3398-2

**Published:** 2018-05-11

**Authors:** Tilahun Tewabe, Selamsew Kindie

**Affiliations:** College of Medicine and Health Science, Bahir Dar University, Bahir Dar, Ethiopia

**Keywords:** Insulin adherence, Level, Felege Hiwot Referral Hospital, Bahir Dar, Ethiopia

## Abstract

**Objectives:**

The objective of this study was to know the level of insulin adherence and to identify factors affecting insulin adherence among diabetes mellitus patients in Felege Hiwot Referral Hospital, Bahir Dar, Northwest Ethiopia.

**Results:**

Prevalence of insulin adherence was 59.2%. Patients who are married [AOR = 0.3 (0.14–0.7)], have regular health care visit [AOR = 3.3 (1.5–7.5)] and accessing insulin with low cost [AOR = 2.9 (1.3–6.3)] were more likely to adhere insulin therapy than their counterparts. Recommendations to increase insulin adherence were: government and non-governmental organizations, volunteers and concerned bodies should support syringe and needles for diabetes patients, health care providers and responsible bodies should give intensive health education about the effect of stopping insulin medication.

## Introduction

Globally, prevalence of people living with diabetes (DM) and is growing in all regions. In 2014 about 422 million (8.5%) peoples had diabetes, compared to 108 million (4.7%) in 1980 [1]. From these more than 14 million people are in Africa and by 2040 this figure will be more than double [2].

DM is the commonest non-communicable disease which is responsible for number of serious health problems and complications in Ethiopia. Like many other developing countries, in Ethiopia too, more attention is given to the communicable diseases such as, HIV/AIDS, tuberculosis and malaria. However, current studies indicated that non communicable diseases like DM became major public health problems in the country [3].

In Ethiopia national data on prevalence and incidence of diabetes mellitus are lacking. However, patient attendance rate, insulin therapy and admission rates in major hospitals are rising. The WHO estimated number of diabetes care in Ethiopia was 800,000 by the year 2000, and this number is expected to increase to 1.8 million by 2030 [4].

Diabetes mellitus is a challenging disease to manage successfully. It has been reported that non adherence rates for chronic illness regimens and lifestyle changes was about 50% and patients with diabetes mellitus are prone to substantial regimen adherence problems [5].

Non adherence to prescribed insulin therapy has been and continues to be a major problem in the world. As any chronic disease non adherence to insulin therapy has been described as taking less than 80% of the prescribed treatment which ranges from 23 to 77% [6].

Some studies in Ethiopia on non adherence rate to treatment on diabetes mellitus patients showed: 70.9% in Jimma University teaching hospital [7], 64.7% in Gonder [8], 33.1% in Tikur Anbassa Hospital [9] showed of diabetes patients had poor adherence to diabetic treatments and another study on patient perception on diabetic patients showed concerns were perceived and experienced adverse effects, inconveniences in handling the medications and access [10].

Adherence to insulin therapy is the major factor with respect to treatment success rates. Poor adherence to insulin therapy may have serious long term and detrimental effect to the patient [11].

These non adherence to insulin medication is a common problem in developing countries like Ethiopia, where economic instability and inadequate access to health care facilities is common [12].

Therefore, the purpose of this study was to assess the level of insulin adherence and its associated factors in Felege Hiwot Referral Hospital (FHRH), Bahir Dar, Northwest Ethiopia.

## Main text

### Methods

#### Study area and setting

Institution based cross sectional study was conducted from March 27 to June 12 2017. The study was conducted in Felege Hiwot Referral Hospital (FHRH) which is found in Bahir Dar city, northwest of Ethiopia. Bahir Dar is the capital city of Amhara regional state. It is 564 kms away from Addis Ababa. The population of the town is 318,429. The town has a total of 17 kebeles. FHRH is the main referral hospital in the region. It provides obstetrics, paediatrics, internal medicine, ophthalmology, general surgery, gynaecology and orthopaedic surgery services. There is also an emergency and outpatient departments (OPD). The DM clinic is situated inside the outpatient department.

#### Sample size determination and sampling procedure

The sample size was calculated using single population proportion formula by considering the following assumptions; proportion (p) of insulin adherence which is 61% [4], 95% level of confidence margin of error (d) = 5, 10% non-response rate. Since the total population is less than 10,000, the sample size was adjusted using correction formula. The final sample size became 182.

To select study participants consecutive sampling technique was used, all diabetes mellitus patients who fulfil the inclusion criteria and coming for follow up at the time of data collection were included until the required sample size is full filled.

### Variables

#### Dependent variables

Adherence to insulin therapy.

#### Independent variable

Sociodemographic factors such as; age, sex, religion, ethnicity, educational level, income, marital status, support system, insulin availability, etc.

#### Operational definition

*Insulin adherence* has been described as taking above 80% of the prescribed treatment and/or agrees between the patient and the health care provider for the last 1 month.

*Insulin non adherence* has been described as taking less than 80% of the prescribed treatment by omitting insulin medication without health personnel order in the last 1 month.

#### Data collection procedure and data quality control

The data was collected by using interviewer administered structured questionnaires which was adopted and modified from previous studies done on similar topics [13]. The questionnaire was first prepared in English and translated to Amharic local language for data collection and pretested on 5% sample size in other hospital and adjustments were done based on the result of the pretest.

Two diploma nurses were recruited as data collectors and one Bachelors of Science nurse was recruited as supervisor. All data collectors and supervisor attended a 1 day training course on the study protocol and interviewing skills.

#### Statistical analysis

The collected data was checked manually for completeness and consistencies, and then it was coded and entered in EPI Info version 3.5.3 and exported to SPSS version 16 for analysis. Descriptive statistics were used to summarize the socio-demographic characteristics’ of the study participants and the level of insulin adherence. To identify factors associated with insulin adherence, binary logistic regression analysis carried out. Strength of association was measured using odds ratio, and 95% confidence intervals. Statistical significance was declared at P value < 0.05.

### Result

#### Socio demographic characteristics

A total of 182 diabetes mellitus patients were participated in this study, which made a response rate of 100%. Most 89 (48.9%) participants were between 31 and 55 years of age. In this study, 100 (54.9%) were males and 82 (45.1%) were female. Regarding residence, most 129 (70.9%) of the respondents were lived in urban. Most participants 156 (85.5%) were Amhara by ethnicity. More than half 114 (62.6%) participants were married and out of them 104 (57.2%) were secondary school and above educated. From total respondents of this study, majority 109 (59.9%) of the respondents had average monthly income of 500–1000 Ethiopian Birr (Table 1).

**Table 1:** Socio-demographic distribution of diabetes patients in Felege-Hiwot Referral Hospital, North West Bahir Dar, Ethiopia, 2016 (n = 182)

Variable (n = 182)	Response	Frequency	Percent
Age	< 30	61	33.5
	31–55	89	48.9
	> 56	32	17.6
Sex	Male	100	54.9
	Female	82	45.1
Residence	Urban	129	70.9
	Rural	53	29.1
Ethnicity	Amhara	156	85.77
	Others^a^	26	14.23
Religion	Orthodox	129	70.9
	Others	53	29.1
Marital status	Not married	115	63.2
	Married	67	36.8
Level of education	Uneducated	78	42.8
	Educated	104	57.2
Occupation	Unemployed	101	55.5
	Employed	81	44.5
Monthly income	< 500	33	18.1
	500–1000	109	59.9
	> 1000	40	22.0

^a^Tigray, agew, oromo

#### Insulin adherence and related characteristics

Greater than half 114 (62.6%) of participants lived with diabetes mellitus for less than 5 years. Most 169 (92.9%) of participants dosing schedule of insulin was twice a day, and more than half 98 (53.8%) of participants visit health care providers once in 3 months.

Majority 176 (96.7%) of the participants did not take their insulin at the same time daily. About 155 (85.2%) respondents took insulin treatment before meal. From total respondents, 109 (59.9%) took their insulin treatment correctly. Whereas 73 (40.1%) defaulted their insulin medication. The reasons for stopping insulin treatment is due to forgetting in 86 (47.3%) study participants, about 23 (12.6%) study participants miss their dose of insulin purposely among them 2 (1.1%) of the respondents can’t afford the cost of insulin, 4 (2.2%) of the respondents miss their insulin treatment due to nature of their work, 6 (3.3%) of the respondents miss their insulin because they dislike the therapy, and 6 (3.3%) miss their treatment due to other reasons like religious practices. About 42 (23.1%) of the respondents stop their medication if they feel better, while 21 (11.5%) of the study participants stop their medication when they feel worse. Other reasons of the respondents stop their insulin treatment includes: medication is expensive 6 (3.3%), take too many medications 8 (4.4%), the medication is complicated 4 (2.2%), inconvenience 4 (2.2%), not sure of the insulin is beneficial 9 (4.9%) and other reasons like preferring holy water than insulin 25 (13.7%) (Table 2).

**Table 2:** Distribution of diabetes patient by duration with diabetic and insulin self-administration in Felege-Hiwot Referral Hospital, North West Bahir Dar, Ethiopia, 2016 (n = 182)

Variable	N = 182	Frequency	Percent
Duration of being diabetic (years)	< 5	114	62.6
	> 5	168	37.4
Duration of insulin therapy	1–4 years	141	77.5
	5 years and above	41	22.5
Dosing schedule of insulin	Once a day	7	3.8
	Twice a day	169	92.9
	More than 2 times	6	3.3
How often do you visit healthcare provider	Once in a month	74	40.7
	Once in 3 months	98	53.8
	Once in 6 months	10	5.5
Time of using needle	Once	15	8.2
	Twice	15	8.2
	2–6 days	100	54.6
	7 and above	52	28.6
From where you bring your insulin	Purchase	134	73.6
	Free	48	26.4
Do you purchase insulin and syringe	Yes	134	73.6
	No	48	26.4
How you rate coast of insulin	Cheap	61	33.5
	Costly	121	66.5
Have regular follow up	Yes	166	91.2
	No	16	8.8
Chronic condition you have	Hypertension	47	25.8
	Heart disease	20	11
	Lung disease	9	4.9
	Other chronic disease	36	19.8
	None	70	38.5
When you took insulin	Before meal	155	85.2
	After meal	27	14.8
Do you take insulin regularly	Yes	109	59.9
	No	73	40.1
Adverse reaction at injection site	No	129	70.9
	Yes	53	29.1
Do you discard insulin after 1 month opening	Yes	168	92.3
	No	14	7.7
Carry insulin out of home	Yes	153	84
	No	29	16

#### Factors associated with insulin adherence

To identify factors associated with insulin adherence, first variables were tested using bivariate analysis. Variables which were associated in the bivariate analysis (P < 0.25) were: sex, marital status, duration of disease, health care visit, cost of insulin, presence of chronic illness, dosing schedules, ability of carrying insulin out of home. Variables which were associated in the bivariate analysis were tested in the final multivariate logistic regression analysis.

After adjusting for potential confounders in the multivariate logistic regression analysis: marital status, health care visit and cost of insulin remained significant.

Marital status of the participants was significantly associated with insulin adherence. Participants who were not married were 0.3 times higher to adhere insulin treatment than those who were married [AOR = 0.3 (0.1–0.7)].

Health care visit of the participants was significantly associated with insulin adherence. Participants who were visiting health care providers once every month were 3.3 times higher to adhere insulin treatment than others [AOR = 3.3 (1.5–7.5)].

Pertaining to cost of insulin, participants who got insulin with free or cheap price were 2.9 times higher to adhere insulin treatment than those who got high cost [AOR = 2.9 (1.3–6.3)] (Table 3).

**Table 3:** Distribution of practice of participants regarding taking their medicine in Felege-Hiwot Referral Hospital, North West Bahir Dar, Ethiopia, 2016 (n = 182)

Variables	Insulin adherence				
	Yes (N and %)	No (N and %)	COR (95% CL)	AOR (95% CL)	P-value
Sex					
Male	53	47	0.524 (0.285–0.962)	–	–
Female	56	26	1	–	–
Marital status					
Not married	57	58	0.283 (0.143–0.560)	*0.309 (0.143–0.670)*	*0.003*
Married	52	15	1	–	–
Duration of the disease					
Up to 5 years	94	47	3.467 (1.678–7.162)	–	
Greater than 5 years	15	26	1	–	
Health care visit					
Once in a month	45	29	0.614 (0.338–1.116)	*3.337 (1.487–7.490)*	*0.003*
More than a month	64	44	1	–	–
Insulin cost					
Free	14	47	3.195 (1.594–6.402)	*2.882 (1.319–6.298)*	*0.008*
Purchase	59	62	1	1	
Other chronic illness					
Yes	71	41	1.459 (0.794–2.677)	–	
No	38	32	1	–	
Some times daily					
Yes	108	68	7.941 (0.908–69.44)	–	
No	1	5	1	–	
Carry insulin out of home					
Yes	95	58	1.755 (0.790–3.899)		
No	14	15	1		

Italic values indicate significant variables

### Discussion

In this study the level of adherence to insulin was 59.9% which is similar studies conducted in Adama 61% [4] and Jimma (61%) [14]. This finding is greater than results found in Sudan (45.6%) [15] Turkey (55.7%) [16]. However it is lower than the study done in Addis Ababa 66.8% [17]. The difference might be due to methodological variations between studies and differences in socio cultural, economical, health and health service utilization and perceptions regarding the importance of good adherence, drug availability characteristics between respondents of the referenced areas and this study.

In this study marital status of respondents was significantly affected level of adherence to insulin therapy. Which is similar to studies done in Adama [4] and Turkey [15]. This may be due to the fact that mirage adds accountability for self by decreasing forgetfulness, ignorance to treatment and gives social support from husband/wife as well from relatives and friends.

Respondents who have regular health care follow up in every month had good adherence than those who have irregular follow up. Regarding to health care visit: 74 (40.7%) had follow up once in a month, 98 (53.6%) had follow up once in 3 month and 10 (5.5%) had follow up once in 6 month.

Cost of insulin was significantly associated with level of adherence to insulin therapy. Which is similar to studies done in Jimma [14] and Iran [18]. This is due to the fact that most of the study participants are poor who have less than 500 birr income per month which can not cover the cost for living, housing and buying medications.

Regarding the schedule of insulin, the most common frequency was twice a day 169 (92.9%). Which is similar with a study done in Iran 65% of respondents practiced twice a day. This discrepancy might happen due to management protocol and socioeconomic differences.

Regarding frequency of using a needles, about 54.9% study participants reused a single needle for 2–6 days, 52 (23.6%) used a single needle for seven or more days, 15 (8.2%) respondents used a single needle once as recommended and the rest 15 (8.2%) respondents used a single needle twice a day. The same study in Iran [18] showed that most of the respondents reused insulin needle more than once. The practice of using needle as recommended deferred in these two study areas. This might be due to lack of knowledge and differences in socio-economic status between study areas.

In this study some respondents 53 (29.1%) developed adverse reaction to insulin injection. The study in Spain [19] about 64.3% of the respondents develop adverse reactions at the injection site. This might be due to lack of adequate knowledge regarding necessity of rotation of site of injection or careless of changing of injection site or due to skin vulnerability to climate changes.

About 153 (84%) respondents in this study carry insulin out of home. A study done in Malaysia [20] showed about 68.9% of the respondents carry their insulin out of home. So this indicates slight difference to our research. This might be due to nature of job socio economic differences between the countries.

In this study, about 168 (92.3%) study participants discard insulin opened after 1 month whereas 14 (7.7%) of the respondents use insulin opened after 1 month this might be due to the thought that the insulin will be expired or decreased its potency.

## Conclusion

In this study level of adherence to insulin therapy was 59.9%. Marital status, regular health care visit and cost of insulin were determinant factors for insulin adherence in the study area. Recommendations to increase adherence to insulin therapy were: government and non-governmental organizations should support syringe and needles for diabetes patients, health care provider and responsible bodies should give intensive health education about the importance of adherence to insulin therapy and the effect of non adherence to insulin treatment.

## Limitations

A limitation of this study is that only the quantitative aspects of insulin therapy adherence was assessed. Since the study is a descriptive study it has a limitation to find additional determinant factors of insulin therapy.

## Abbreviations

BDU: Bahir Dar University
AOR: adjusted odds ratio
CL: confidence level
DM: diabetes mellitus
SD: standard deviation
WHO: World Health Organization

## References

[1] WHO, World Health Day 2016. WHO, call for global action to halt rise in and improve care for people with diabetes. WHO; 2016.

[2] International Diabetes Federation. 2015.

[3] World R (2013). Diabetes and associated disease from Ethiopian perspective; systemic review. Ethiop J Health Dev..

[4] Belayneh K, Abebaw W (2013). Non-adherence and contributing factor among ambulatory patients with anti-diabetic medication in Adama Referral Hospital. J Diab Res..

[5] Eman M, Saridi B, Gererd L (2011). Compliance to diabetes self-management in rural El-minia, Egypt. Cent Eur J Public Health.

[6] Peyrot M, Barnett H, Wored K (2012). Insulin adherence behaviours and barriers in the multi-national global attitudes of patient and physicians in insulin therapy. Diab Med..

[7] Kassahun T, Eshetie T, Gesesew H (2016). Factors associated with glycemic control among adult patients with type 2 diabetes mellitus: a cross-sectional survey in Ethiopia. BMC Res Notes.

[8] Abebe SM, Berhane Y, Worku A, Alemu S, Mesfin N (2015). Level of sustained glycemic control and associated factors among patients with diabetes mellitus in Ethiopia: a hospital-based cross-sectional study. Diab Metabol Syndr Obes Targets Ther.

[9] Gerada Y, Mengistu Z, Demessie A, Fantahun A, Gebrekirstos K (2017). Adherence to insulin self administration and associated factors among diabetes mellitus patients at Tikur Anbessa specialized hospital. J Diab Metabol Disord.

[10] Habte BM, Kebede T, Fenta TG, Boon H (2017). Ethiopian patients’ perceptions of anti-diabetic medications: implications for diabetes education. J Pharm Policy Pract.

[11] Tamiru S, Alemseged F (2010). Risk factors for cardiovascular diseases among diabetic patients in southwest Ethiopia. Ethiop J Health Sci..

[12] Konistantinos P, Nikolaos P, Macieje B, Dimirtios P (2016). Complication of diabetes 2016. J Diab Res..

[13] Yusuf G (2014). assessment of adherence to insulin self-administration and associated factors among diabetes mellitus patient at endocrinology unit of TASH.

[14] ADA (2017). Standards of medical care in diabetes. Am Diab Assoc.

[15] Tarig M (2016). Factors affecting medication adherence in type 2 sudanese diabetic patient. Pharmacol Pharm.

[16] Gogas D, Ozcan S, Deyneli O (2015). adherence to insulin treatment in insulin naive type 2 diabetes patients initiated on different insulin regime. Patient Preference Adherence..

[17] Tsehay T, Engidawork E, Ahmed A (2016). Assessment of antidiabetic medication adherence and Itsdeterminants among ambulatory patients with type 2 diabetes at Tikur Anbessa specialized hospital, AddisAbaba, Ethiopia. J Pharm Altern Med..

[18] Shady J, Gerd H, Boint K (2014). Insulin adherence in patient with diabetes. Prim Care Diab..

[19] Hasen B (2012). Evidence-based clinical guidelines for injection of insulin for adults with diabetes mellitus clinical nursing specialist.

[20] Jalan R (2014). Diabetes knowledge and medication adherence among patient with type 2 DM. Malay.

